# Heart rate variability and atrial fibrillation in the general population: a longitudinal and Mendelian randomization study

**DOI:** 10.1007/s00392-022-02072-5

**Published:** 2022-08-13

**Authors:** Sven Geurts, Martijn J. Tilly, Banafsheh Arshi, Bruno H. C. Stricker, Jan A. Kors, Jaap W. Deckers, Natasja M. S. de Groot, M. Arfan Ikram, Maryam Kavousi

**Affiliations:** 1 Department of Epidemiology, Erasmus MC, University Medical Center Rotterdam, Rotterdam, The Netherlands; 2 Department of Medical Informatics, Erasmus MC, University Medical Center Rotterdam, Rotterdam, The Netherlands; 3 Department of Cardiology, Erasmus MC, University Medical Center Rotterdam, Rotterdam, The Netherlands

**Keywords:** Atrial fibrillation, Epidemiology, Heart rate variability, Mendelian randomization, Risk factors, Sex differences

## Abstract

**Background:**

Sex differences and causality of the association between heart rate variability (HRV) and atrial fibrillation (AF) in the general population remain unclear.

**Methods:**

12,334 participants free of AF from the population-based Rotterdam Study were included. Measures of HRV including the standard deviation of normal RR intervals (SDNN), SDNN corrected for heart rate (SDNNc), RR interval differences (RMSSD), RMSSD corrected for heart rate (RMSSDc), and heart rate were assessed at baseline and follow-up examinations. Joint models, adjusted for cardiovascular risk factors, were used to determine the association between longitudinal measures of HRV with new-onset AF. Genetic variants for HRV were used as instrumental variables in a Mendelian randomization (MR) analysis using genome-wide association studies (GWAS) summary-level data.

**Results:**

During a median follow-up of 9.4 years, 1302 incident AF cases occurred among 12,334 participants (mean age 64.8 years, 58.3% women). In joint models, higher SDNN (fully-adjusted hazard ratio (HR), 95% confidence interval (CI) 1.24, 1.04–1.47, *p* = 0.0213), and higher RMSSD (fully-adjusted HR, 95% CI 1.33, 1.13–1.54, *p* = 0.0010) were significantly associated with new-onset AF. Sex-stratified analyses showed that the associations were mostly prominent among women. In MR analyses, a genetically determined increase in SDNN (odds ratio (OR), 95% CI 1.60, 1.27–2.02, *p* = 8.36 × 10^–05^), and RMSSD (OR, 95% CI 1.56, 1.31–1.86, *p* = 6.32 × 10^–07^) were significantly associated with an increased odds of AF.

**Conclusion:**

Longitudinal measures of uncorrected HRV were significantly associated with new-onset AF, especially among women. MR analyses supported the causal relationship between uncorrected measures of HRV with AF. Our findings indicate that measures to modulate HRV might prevent AF in the general population, in particular in women.

**Graphical abstract:**

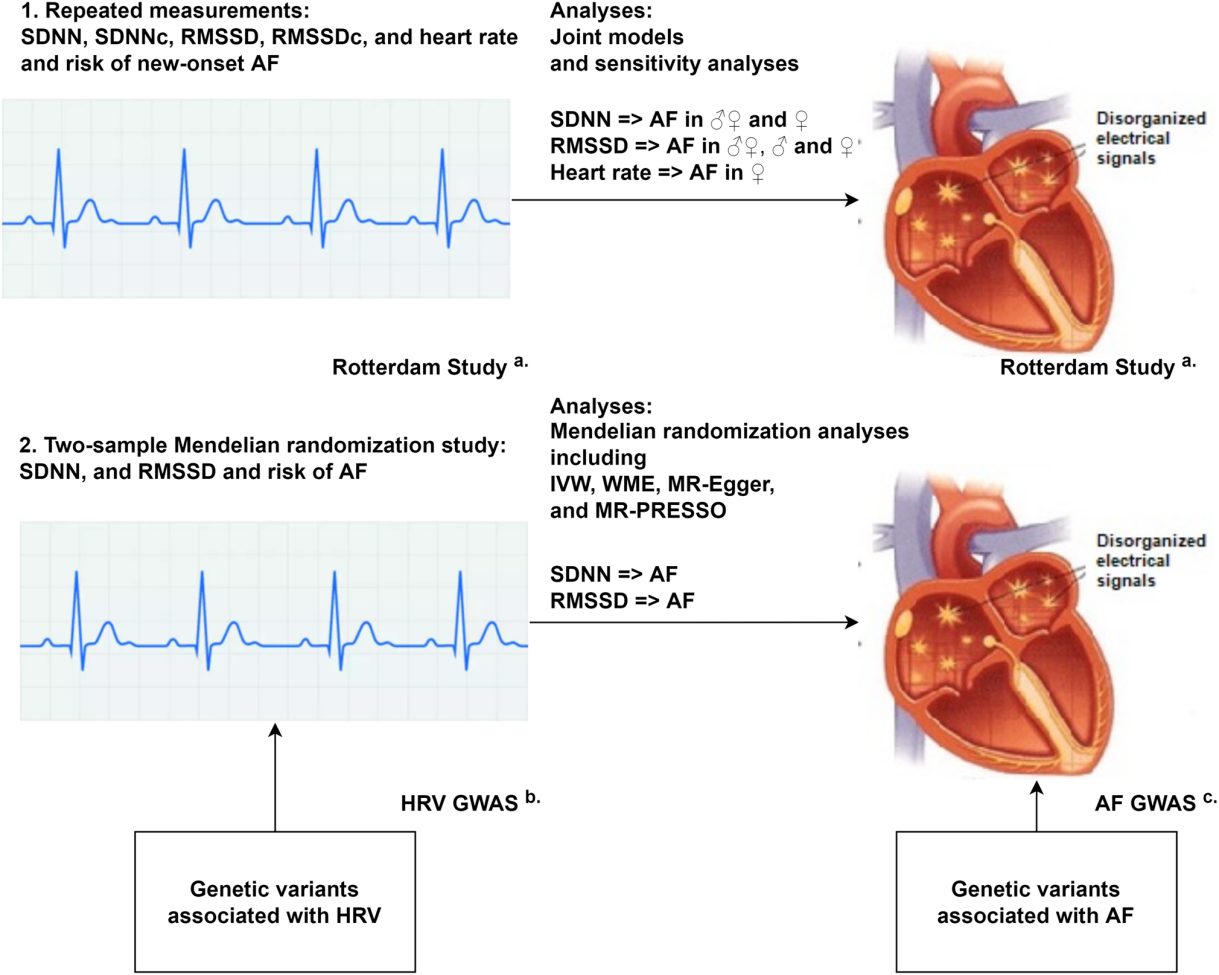

*AF*; atrial fibrillation, *GWAS*; genome-wide association study, *IVW*; inverse variance weighted, *MR*; Mendelian randomization, *MR-PRESSO*; MR-egger and mendelian randomization pleiotropy residual sum and outlier, *RMSSD*; root mean square of successive RR interval differences, *RMSSDc*; root mean square of successive RR interval differences corrected for heart rate, *SDNN*; standard deviation of normal to normal RR intervals, *SDNNc*; standard deviation of normal to normal RR intervals corrected for heart rate, *WME*; weighted median estimator.

^a^Rotterdam Study *n*=12,334

^b^HRV GWAS *n*=53,174

^c^AF GWAS *n*=1,030,836

**Supplementary Information:**

The online version contains supplementary material available at 10.1007/s00392-022-02072-5.

## Introduction

Atrial fibrillation (AF), the most common cardiac arrhythmia, is associated with substantial morbidity and mortality and represents a significant burden on healthcare [1–4]. The exact AF pathogenesis remains to be identified. It has recently been suggested that cardiac autonomic imbalance could play a role in AF pathophysiology by promoting a decline in cardiac function [5–10].

Heart rate variability (HRV) is considered a non-invasive, accessible measure that may reflect the complex interaction between the autonomic nervous system and the heart [11, 12]. A complex relationship between HRV and AF has been suggested [5–10, 13]. Specifically, lower and higher levels of HRV may lead to decline in cardiac function and subsequently give rise to AF [5–10, 13]. Recent evidence shows that sex differences with regard to AF burden, pathophysiology, and prognosis exist [14]. However, the previous observational studies have been limited to either a cross-sectional design or a single measurement of HRV and did not evaluate sex differences. In addition, observational studies are prone to residual confounding and reverse causality [15].

Genome-wide association studies (GWAS) have identified genetic variants/single nucleotide polymorphisms (SNPs) for multiple assessments of HRV [16] and AF [17, 18]. Pathway and tissue enrichment analyses suggest that HRV SNPs are preferentially expressed within the sinoatrial node [16]. Moreover, AF SNPs have been suggested to affect the cardiac ion channels, cardiac calcium signaling, and the heart and skeletal muscles [17, 18]. This suggests that there may be a genetic foundation underlying the association between HRV and AF.

We aimed to investigate the association between longitudinal measures of HRV and heart rate with the risk of new-onset AF in the general population. Additionally, we used a comprehensive Mendelian randomization (MR) analysis using summary-level data from GWAS on measures of HRV and AF to investigate the potential causal relationship between HRV and AF.

## Methods

### Study population

The current study was embedded within the Rotterdam Study [19, 20]. The Rotterdam Study is a prospective population-based cohort study that aims to assess the occurrence and progression of risk factors for chronic diseases in middle-age and elderly persons. During 1990–1993, all inhabitants of the Ommoord district in the city of Rotterdam in The Netherlands aged ≥ 55 years were invited for the study. A total of 7983 (78% of all invitees) agreed to participate (RS-I). In 2000, the cohort was extended with 3011 participants who had become ≥ 55 years or had migrated into the research area (RS-II). In 2006, the cohort was again extended with 3932 participants who were ≥ 45 years (RS-III). The overall response rate at baseline was 72%. Participants attended follow-up examinations every 3–5 years. Outcome data on morbidity and mortality were continuously collected through linkage with digital files from general practitioners in the study area [19, 20].

For the present study, we included participants at study entry of the three recruitment waves. Participants with prevalent AF at baseline (*n* = 559), no informed consent for follow-up data collection (*n* = 305), no follow-up time (*n* = 6) or no measures of HRV (*n* = 1722) were excluded. Among the 12,334 free of AF included participants, 12,334 had at least 1 measurement for standard deviation of normal RR intervals (SDNN), SDNN corrected for heart rate (SDNNc), RR interval differences (RMSSD), RMSSD corrected for heart rate (RMSSDc), and heart rate. 8,832 participants had 2 measurements, 3837 had 3 measurements, 1817 had 4 measurements, and 787 participants had 5 measurements that were available during follow-up (before date of onset of AF, date of death, loss to follow-up, or to January 1, 2014, whichever occurred first).

### Assessment of heart rate variability

Participants underwent a 10-s 12-lead resting electrocardiogram (ECG) using an ACTA Gnosis IV ECG recorder (Esaote Biomedica, Florence, Italy) and the ECG records were digitally stored. Subsequently, Modular ECG Analysis System (MEANS) was used to interpret the ECGs [21]. ECGs of individuals with a pacemaker, < 5 RR intervals between normal beats or > 5 premature supra- and/or ventricular complexes were excluded for the assessment of HRV [22]. In addition, the remaining ECGs marked as non-sinus arrhythmia and sinus arrhythmia by MEANS were manually assessed by 2 medical doctors to rule out and exclude atrial fibrillation/flutter, other arrhythmias, and ECGs with poor signal quality. Sinus rhythm (including sinus arrhythmia) is based on the detection by MEANS of regular P waves that have a fixed coupling interval with the following QRS complexes [21]. Furthermore, a random sample of 200 ECGs marked as sinus rhythm by MEANS were also manually checked by 2 medical doctors and 199 ECGs were found to be in sinus rhythm during the manual assessment indicating a very high positive predictive value of MEANS which was also demonstrated in earlier work [21]. RR intervals between 2 adjacent normal beats were used to compute the mean heart rate and time-domain indices of HRV; SDNN and RMSSD. Moreover, as HRV is potentially inversely and exponentially associated with heart rate, we additionally used heart rate corrected values of RMSSD (RMSSDc), and SDNN (SDNNc) using an exponential model [22–26]. The reproducibility of the HRV data was evaluated in a later cohort of the Rotterdam Study, in which ECG recordings of 3–5 min were made. From a sample of 310 3–5 min ECGs, we extracted from each recording two 10-s ECGs, one after the first minute of recording, the second after 2 minutes. The sample of 310 pairs of 10-s ECGs was also manually assessed by 2 medical doctors to rule out and exclude arrhythmias, and ECGs with poor signal quality. After exclusion, 211 ECG pairs remained and were used to calculate the HRV measures. Differences were examined using the paired T-test. The HRV measures did not statistically significantly differ from each other (*p* = 0.087 for RR, *p* = 0.415 for SDNN, and *p* = 0.427 for RMSSD).

### Assessment of atrial fibrillation

AF was defined in accordance with the European Society of Cardiology (ESC) guidelines [4]. The methods on event adjudication for prevalent and incident AF within the Rotterdam Study have been described in detail earlier [20]. In short, AF was assessed at baseline and follow-up examinations using a 10-s 12-lead ECG with an ACTA Gnosis IV ECG recorder (Esaote Biomedica, Florence, Italy). The ECG records were then stored digitally and analyzed with MEANS [21]. Thereafter, 2 medical doctors validated the diagnosis of AF and in case of disagreement a cardiologist was consulted [3]. Additional follow-up data were obtained from medical files of participating general practitioners, hospitals, outpatient clinics, national registration of all hospitals discharge diagnoses, and follow-up examinations at the research center. The date of incident AF was defined as the date of the first occurrence of symptoms suggestive of AF with subsequent ECG verification obtained from the medical records. Participants were followed from the date of enrollment in the Rotterdam Study until the date of onset of AF, date of death, loss to follow-up, or to January 1, 2014, whichever occurred first.

### Assessment of cardiovascular risk factors

The cardiovascular risk factors included in this study were body mass index (BMI), total cholesterol, high-density lipoprotein cholesterol, hypertension, smoking status, history of diabetes mellitus, history of coronary heart disease, history of heart failure, left ventricular hypertrophy on the ECG, use of cardiac medication, use of antihypertensive medication, use of beta blockers, use of calcium blockers, and use of lipid lowering medication. Methods for measurements of cardiovascular risk factors are explained in detail in the Methods S1 [3, 19, 20].

### Selection of genetic variants on heart rate variability and atrial fibrillation

Genetic variants associated with HRV were used as instrumental variables for the MR analyses. The genetic variants were retrieved from publically available summary statistics from 2 GWAS [16–18]. Details regarding these study populations are depicted in Tables S4 and S5. For HRV, we retrieved independent genetic variants from a GWAS on HRV that assessed SDNN and RMSSD as log transformed continuous measures. This GWAS meta-analysis on HRV included 53,174 participants from European descent [16]. In addition, we retrieved independent genetic variants that were associated with AF from a GWAS that included 1,030,836 European participants (60,620 AF cases and 970,216 controls) [17]. We only included independent genetic variants in the subsequent MR analyses (*p*-value < 5.0 × 10^–08^ genome-wide significant and *r*^2^ < 0.1).

### Statistical analyses

#### Joint model analyses

The baseline characteristics of the study population are presented as mean with standard deviation (SD) or number (n) with percentages as appropriate. Differences between men and women were examined by Student’s *T*-test (normal distribution) or the Mann Whitney *U*-test (skewed distribution) for continuous variables, and Chi-Square Test for categorical variables. The distributions of the different HRV measures and heart rate were skewed. Therefore, a natural logarithmic transformation was used to obtain a normal distribution.

**Fig. 1:**
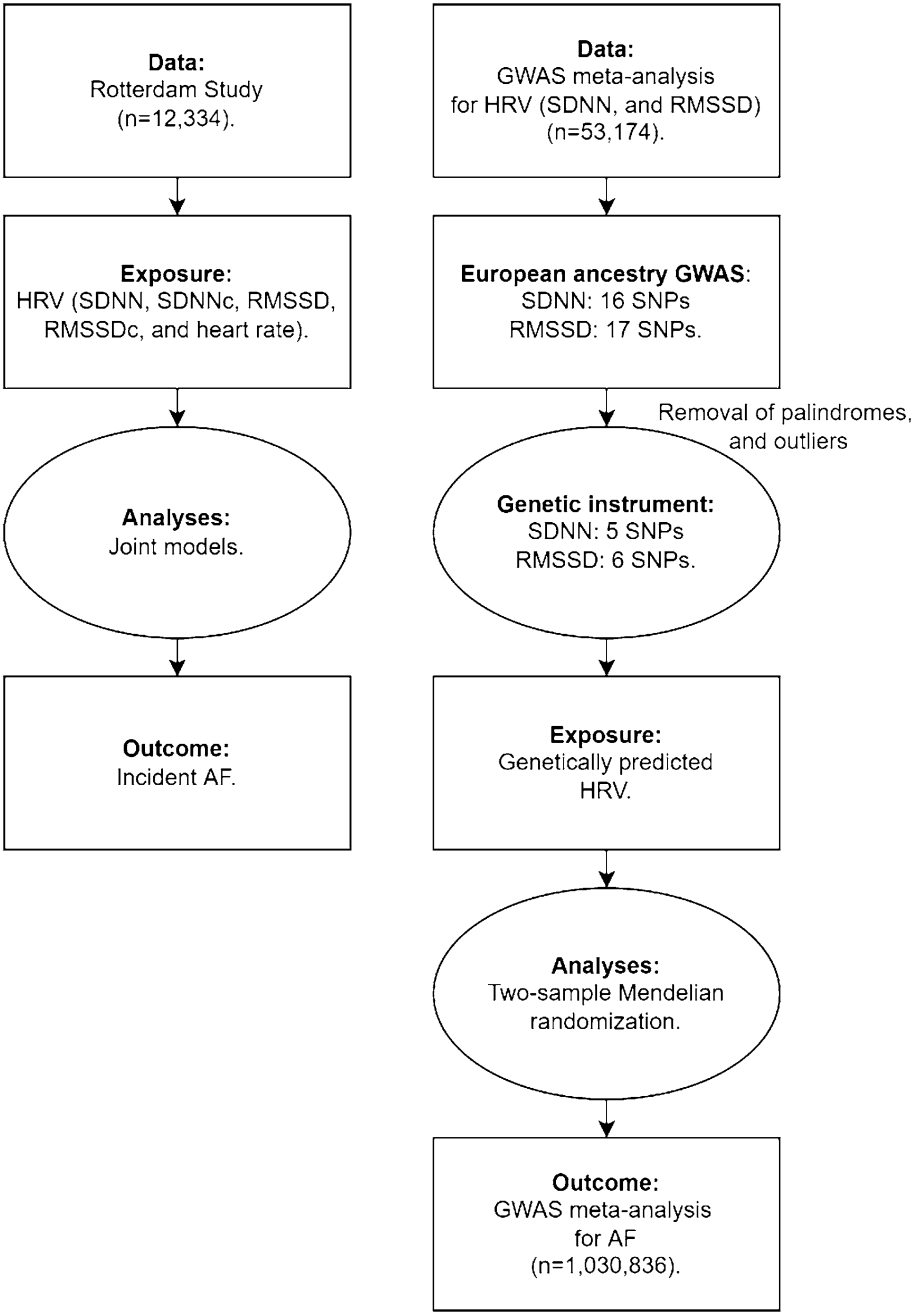
Flow chart for the conducted analyses. Abbreviations: AF, atrial fibrillation; GWAS, genome-wide association study; HRV, heart rate variability; SDNN, standard deviation of normal to normal RR intervals; SDNNc, standard deviation of normal to normal RR intervals corrected for heart rate; RMSSD, root mean square of successive RR interval differences; RMSSDc, root mean square of successive RR interval differences corrected for heart rate; SNPs, single-nucleotide polymorphisms

Competing risk analyses were employed using joint models for longitudinal and time-to-event data. To investigate the association between longitudinal measures of HRV with the risk of new-onset AF with mortality as a competing event, cause-specific hazard ratios (HRs) with their 95% confidence intervals (CIs) were calculated to quantify the associations (Fig. 1). See the Methods S2 for more details on the rationale, imputation and sensitivity analyses of the joint model analyses [27, 28].

The analyses were done in the total study population and for men and women separately. Additionally, we reported the *p*-values of sex interaction from the joint model. All models (mixed- and survival models) were adjusted for age, sex (if applicable), and cohort (model 1), and additionally for cardiovascular risk factors including body mass index, total cholesterol, high-density lipoprotein cholesterol, hypertension, smoking status, history of diabetes mellitus, history of coronary heart disease, history of heart failure, left ventricular hypertrophy on the ECG, cardiac medication, beta blockers, calcium blockers, and use of lipid lowering medication (model 2). Time was measured in years after baseline and the variables from model 1 and 2 were treated as covariates in the subsequent models.

#### Mendelian randomization analyses

We conducted two-sample MR analyses to examine the potential causal association between HRV and AF. The inverse variance weighted (IVW) method is the main method used in our analyses [29]. MR estimates were presented as odds ratios (ORs) with corresponding 95% CIs (Fig. 1). See the Methods S3, for more details on the rationale, assumptions and sensitivity analyses of the MR analyses [15, 29–35].

A two-tailed *p* < 0.05 was considered statistically significant. The data management was done using IBM SPSS Statistics version 25.0 for Windows (IBM Corp, Armonk, New York). The statistical analyses were done using the R packages “JMbayes2”, [36] and “TwoSampleMR”(30, 34, 35) in R software (R 4.0.2; R Foundation for Statistical Computing, Vienna, Austria) [37].

## Results

### Joint model analyses

A total of 12,334 participants, 5140 men (41.7%) and 7194 women (58.3%), were eligible for the analyses. Baseline characteristics for the total study population and stratified by sex are presented in Table 1. The mean age of the total study population was 64.8 ± 9.5 years and 58.3% were women. Median values for SDNN, SDNNc, RMSSD, RMSSDc, and heart rate were 16.2 ms, 27.0 ms, 16.3 ms, 33.7 ms, and 69.0 beats/min, respectively. See Table 1 for more details.

**Table 1:** Baseline characteristics of the total study population and stratified by sex

Baseline characteristics^a^	Total study population *n* = 12,334	Men *n* = 5140	Women *n* = 7,194	*p*-value^d^
Age, years	64.8 ± 9.5	64.0 ± 8.9	65.3 ± 10.0	< 0.001
Women, *n* (%)	7194 (58.3)	NA	7194 (100)	NA
Body mass index, kg/m^2^	27.0 ± 4.1	26.7 ± 3.6	27.2 ± 4.5	< 0.001
Total cholesterol, mmol/L^b^	6.1 ± 1.2	5.9 ± 1.2	6.4 ± 1.2	< 0.001
High-density lipoprotein cholesterol, mmol/L^b^	1.4 ± 0.4	1.2 ± 0.3	1.5 ± 0.4	< 0.001
Systolic blood pressure, mmHg	138.4 ± 21.4	139.0 ± 20.5	138.0 ± 22.1	0.011
Diastolic blood pressure, mmHg	77.7 ± 11.8	78.9 ± 11.8	76.9 ± 11.8	< 0.001
Hypertension, *n* (%)	7225 (58.6)	3010 (58.6)	4215 (58.7)	0.928
Smoking status, *n* (%)				< 0.001
Never	3915 (32.3)	717 (14.1)	3198 (45.3)	
Former	5293 (43.6)	2884 (56.7)	2409 (34.2)	
Current	2931 (24.1)	1484 (29.2)	1447 (20.5)	
History of diabetes mellitus, *n* (%)	1268 (10.3)	595 (11.6)	673 (9.4)	< 0.001
History of coronary heart disease, *n* (%)	756 (6.3)	547 (10.9)	209 (3.0)	< 0.001
History of heart failure, *n* (%)	210 (1.7)	84 (1.6)	126 (1.8)	0.617
Left ventricular hypertrophy, *n* (%)	696 (6.2)	402 (8.7)	294 (4.5)	< 0.001
Cardiac medication, *n* (%)	646 (5.5)	299 (6.2)	347 (5.1)	0.012
Antihypertensive medication, *n* (%)	3344 (28.6)	1345 (27.7)	1999 (29.3)	0.068
Beta blockers, *n* (%)	1591 (13.6)	698 (14.4)	893 (13.1)	0.042
Calcium blockers, *n* (%)	1006 (8.6)	453 (9.3)	553 (8.1)	0.019
Lipid lowering medication, *n* (%)	1274 (10.9)	616 (12.7)	658 (9.6)	< 0.001
SDNN, ms^c^	16.2 (10.3–26.8)	16.2 (10.1–27.5)	16.2 (10.4–26.2)	0.705
SDNNc, ms^c^	27.0 (16.8–45.9)	25.1 (14.9–43.7)	28.3 (18.2–47.6)	< 0.001
RMSSD, ms^c^	16.3 (10.4–26.5)	16.0 (9.9–26.3)	16.4 (10.7–27.0)	0.002
RMSSDc, ms^c^	33.7 (20.5–59.4)	28.7 (17.2–53.3)	36.9 (23.3–63.3)	< 0.001
Heart rate, beats/min^c^	69.0 (62.0–76.7)	67.0 (60.1–75.3)	70.3 (63.6–77.6)	< 0.001

Values are shown before imputation and therefore not always add up to 100%

RMSSD, root mean square of successive RR interval differences; RMSSDc, root mean square of successive RR interval differences corrected for heart rate; SDNN, standard deviation of normal to normal RR intervals; SDNNc, standard deviation of normal to normal RR intervals corrected for heart rate

^a^Values are mean (standard deviation) or number (percentages)

^b^SI conversion factors: To convert cholesterol to mg/dL, divide values by 0.0259

^c^Non-transformed median with interquartile range

^d^Statistical significance for continuous data was tested using the Student’s *T*-test (normal distribution) or the Mann Whitney *U*-test (skewed distribution) and for categorical data was tested using the Chi Square Test

During a median follow-up of 9.4 years (interquartile range (IQR), 6.2–15.1), 1302 incident AF cases (10.6%) (613 in men and 691 in women) and 4004 mortality cases (32.5%) (1,740 in men and 2264 in women) occurred. The incidence rate of AF was 9.6 per 1000 person-years in the total study population (11.5 per 1000 person-years in men, 8.4 per 1000 person-years in women) and the incidence rate of mortality was 29.5 per 1000 person-years in the total study population (32.6 per 1000 person-years in men, 27.5 per 1000 person-years in women).

Joint models showed significant associations in model 2 with the risk of new-onset AF in the total study population for a higher SDNN (HR, 95% CI 1.24, 1.04–1.47, *p* = 0.0213), and a higher RMSSD (HR, 95% CI 1.33, 1.13–1.54, *p* = 0.0010). However, a higher SDNNc (HR, 95% CI 1.06, 0.89–1.23, *p* = 0.4784), higher RMSSDc (HR, 95% CI 1.09, 0.96–1.22, *p* = 0.1774), and a lower heart rate (HR, 95% CI 1.21, 0.74–1.99, *p* = 0.4781) were not significantly associated with the risk of new-onset AF in the total study population. The effect estimates slightly attenuated in model 2 in comparison to model 1, but SDNN, and RMSSD remained significant. See Table 2 for more details.

**Table 2:** Association between longitudinal measures of heart rate variability with the risk of new-onset atrial fibrillation in the total study population and stratified by sex

Heart rate variability measures	Total study population		Men		Women	
	Cause-specific HR (95% CI)					
	Model 1^a^	Model 2^b^	Model 1^a^	Model 2^b^	Model 1^a^	Model 2^b^
SDNN^c^	1.22 (1.02–1.48), *p* = 0.0287	1.24 (1.04–1.47), *p* = 0.0213	1.09 (0.87–1.38), *p* = 0.4659	1.16 (0.92–1.45), *p* = 0.2103	1.46 (1.13–2.03), *p* = 0.0031	1.36 (1.03–1.79), *p* = 0.0278
SDNNc^c^	1.00 (0.86–1.17), *p* = 0.9741	1.06 (0.89–1.23), *p* = 0.4784	1.04 (0.86–1.26), *p* = 0.7112	1.10 (0.90–1.32), *p* = 0.3122	0.98 (0.77–1.27), *p* = 0.8162	1.01 (0.81–1.27), *p* = 0.9274
RMSSD^c^	1.38 (1.20–1.60), *p* < 0.0001	1.33 (1.13–1.54), *p* = 0.0010	1.21 (1.01–1.46), *p* = 0.0392	1.23 (1.01–1.48), *p* = 0.0414	1.60 (1.29–2.00), *p* < 0.0001	1.47 (1.16–1.89), *p* = 0.0018
RMSSDc^c^	1.06 (0.94–1.20), *p* = 0.3302	1.09 (0.96–1.22), *p* = 0.1774	1.10 (0.94–1.28), *p* = 0.2320	1.13 (0.96–1.30), *p* = 0.1411	1.02 (0.85–1.21), *p* = 0.8413	1.04 (0.87–1.23), *p* = 0.6994
Heart rate^c^	1.60 (0.90–2.80), *p* = 0.1083	1.21 (0.74–1.99), *p* = 0.4781	0.77 (0.42–1.40), *p* = 0.3979	0.66 (0.36–1.19), *p* = 0.1643	2.21 (1.13–4.10), *p* = 0.0261	1.88 (1.02–3.67), *p* = 0.0408

CI, confidence interval; HR, hazard ratio; RMSSD, root mean square of successive RR interval differences; RMSSDc, root mean square of successive RR interval differences corrected for heart rate; SDNN, standard deviation of normal to normal RR intervals; SDNNc, standard deviation of normal to normal RR intervals corrected for heart rate

^a^Adjusted for age, sex (if applicable), and cohort

^b^Adjusted for age, sex (if applicable), cohort, body mass index, total cholesterol, high-density lipoprotein cholesterol, hypertension, smoking status, history of diabetes mellitus, history of coronary heart disease, history of heart failure, left ventricular hypertrophy on the electrocardiogram, cardiac medication, beta blockers, calcium blockers, and use of lipid lowering medication

^c^Hazard ratios represent 1 unit increase of ln(SDNN), ln(SDNNc), ln(RMSSD), ln(RMSSDc), and 1 unit decrease of ln(heart rate) with the risk of new-onset atrial fibrillation

The sex-stratified analyses from model 2 showed that in men only the association for a higher RMSSD (HR, 95% CI 1.23, 1.01–1.48, *p* = 0.0414) with the risk of new-onset AF was significant. The analyses in women showed significant associations for a higher SDNN (HR, 95% CI 1.36, 1.03–1.79, *p* = 0.0278), higher RMSSD (HR, 95% CI 1.47, 1.16–1.89, *p* = 0.0018), and lower heart rate (HR, 95% CI 1.88, 1.02–3.67, *p* = 0.0408) with the risk of new-onset AF. See Table 2 for more information. In model 2, the *p*-values of the sex interaction in the joint model for SDNN, SDNNc, RMSSD, RMSSDc, and heart rate were *p* = 0.1077, *p* = 0.7638, *p* = 0.0065, *p* = 0.8465, and *p* = 0.1298, respectively.

All results of the joint model sensitivity analyses are depicted in Results S1.

### Mendelian randomization analyses

A total of 33 genome-wide significant independent genetic variants were associated with HRV represented by SDNN (*n* = 16) and RMSSD (*n* = 17), respectively. A total of 5 SNPs for SDNN and 6 SNPs for RMSSD were available in the AF GWAS and were used for the MR analyses after removal of potential outliers. All individual genetic instruments for SDNN and RMSSD had a *F* statistic > 10 (median for SDNN, 53.2 (IQR, 51.8–70.8) and median for RMSSD, 54.7 (IQR, 43.1–69.3)) and were, therefore, considered to be of sufficient strength to be used in the MR analyses. The effect estimates of the genetic variants associated with SDNN, RMSSD and AF that were used in the MR analyses are presented in Table S4.

**Fig. 2:**
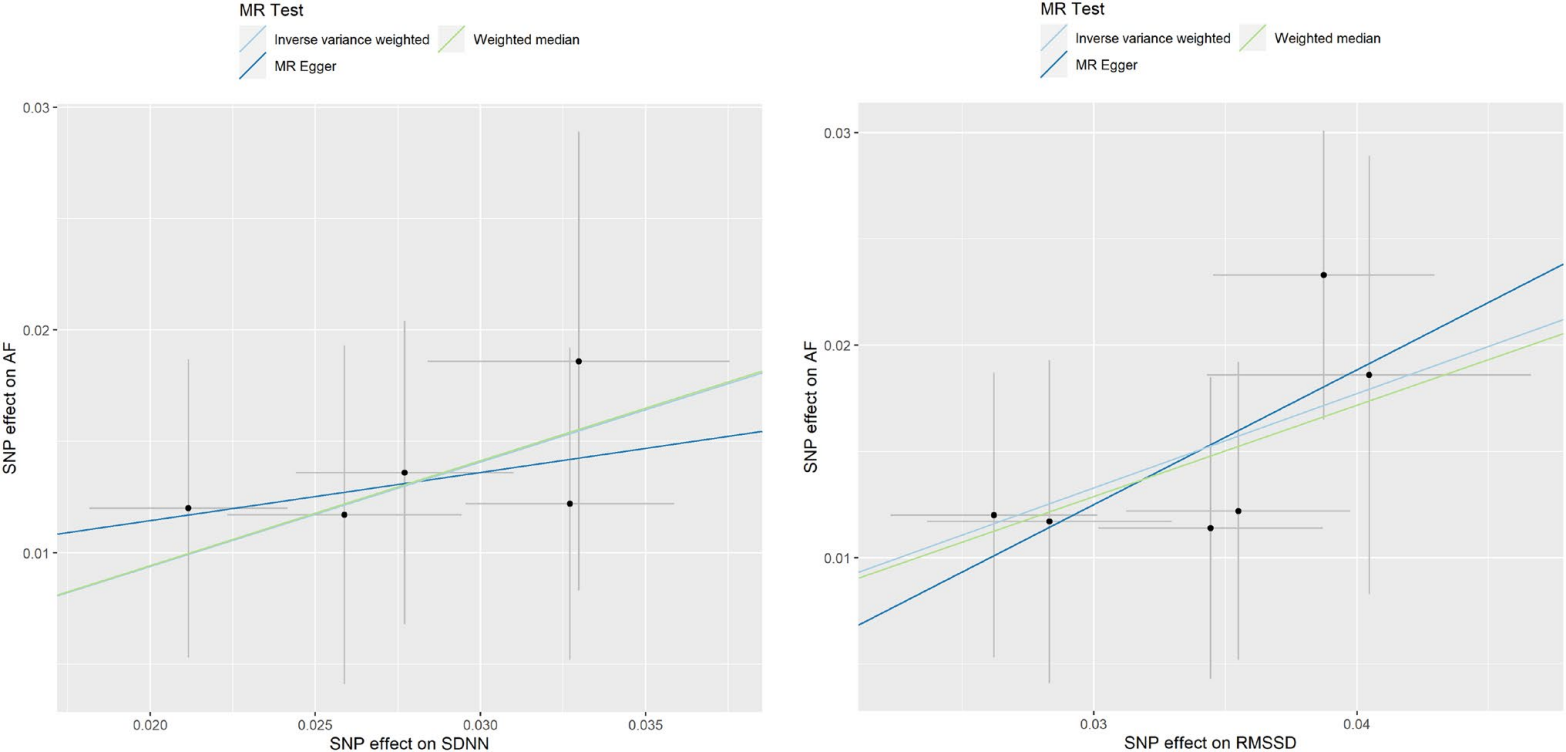
Scatter plot which visualizes the causal effect estimate for each individual genetic variant on SDNN, and RMSSD and its effect on atrial fibrillation. Abbreviations: IVW, inverse variance weighted; RMSSD, the root mean square of successive RR interval differences; SDNN, standard deviation of normal to normal RR intervals; SNPs, single nucleotide polymorphisms; WME, weighted median estimator. The dots represent the corresponding effect estimates of each individual genetic variant. The crosses around the dots represent the 95% confidence intervals of the corresponding effect estimates of each individual genetic variant

The MR estimates from the association between HRV and AF based on the IVW, weighted median estimator (WME), and MR-Egger methods are presented in Table 3. Specifically, MR analyses supported the causal effects of genetically determined SDNN and RMSSD on AF risk (for SDNN: *n* = 5 SNPs, OR, 95% CI 1.60, 1.27–2.02, *p* = 8.36 × 10^–05^ and for RMSSD: *n* = 6 SNPs, OR, 95% CI 1.56, 1.31–1.86, *p* = 6.32 × 10^–07^). A graphical presentation of the results can be found in Fig. 2.

**Table 3:** Mendelian randomization analyses between heart rate variability and atrial fibrillation

Method	SDNN (*n* = 5 SNPs)		RMSSD (*n* = 6 SNPs)	
	OR (95% CI)^a^	*p*-value	OR (95% CI)^a^	*p*-value
IVW	1.60 (1.27–2.02)	8.36 × 10^–05^	1.56 (1.31–1.86)	6.32 × 10^–07^
WME	1.60 (1.22–2.11)	8.28 × 10^–04^	1.54 (1.23–1.92)	1.49 × 10^–04^
MR-Egger slope	1.24 (0.29–5.40)	7.92 × 10^–01^	1.89 (0.58–6.08)	3.48 × 10^–01^
MR-Egger intercept	1.01 (0.97–1.05)	7.55 × 10^–01^	0.99 (0.96–1.03)	7.63 × 10^–01^
Heterogeneity	NA	9.82 × 10^–01^	NA	9.26 × 10^–01^
Global test MR-PRESSO	NA	9.92 × 10^–01^	NA	9.08 × 10^–01^

CI, confidence interval; IVW, inverse variance weighted; MR, Mendelian randomization; MR-PRESSO, Mendelian randomization pleiotropy residual sum and outlier; NA, not applicable; OR, odds ratio; RMSSD, the root mean square of successive RR interval differences; SDNN, standard deviation of normal to normal RR intervals; SNPs, single-nucleotide polymorphisms; WME, weighted median estimator

^a^Odds ratios represent a genetically determined 1 unit increase of ln(SDNN), and ln(RMSSD) with the odds of atrial fibrillation

The results of the MR sensitivity analyses are depicted in Results S2 [33].

## Discussion

Our study shed light on the complex interaction between HRV and AF. Our joint model analyses showed that longitudinal measures of SDNN, and RMSSD were significantly associated with new-onset AF in the general population while SDNNc, RMSSDc, and heart rate were not significantly associated. Sex-stratified analyses showed that RMSSD among men, and SDNN, RMSSD, and heart rate among women were significantly associated with new-onset AF. MR analyses supported the causal association between SDNN, and RMSSD with AF. Our findings indicate that treatment to modulate HRV might prevent AF in the general population, in particular in women.

The exact mechanism that underlies the relationship between HRV and AF remains incompletely understood. Shared underlying risk factors, such as obesity, diabetes mellitus, and coronary heart disease, could influence HRV and are also implicated in AF pathophysiology [1, 5, 12, 38, 39]. In our study, however, the associations of HRV with incident AF slightly attenuated, but remained significant after extensive adjustment for shared cardiovascular risk factors. The increase in left atrial size that has been associated with HRV could suggest a role for HRV in AF pathogenesis that is mediated by the left atrium [13]. Moreover, autonomic imbalance could trigger an inflammatory response that can subsequently lead to AF [12]. Finally, the effect of the GWAS-identified HRV SNPs on the genes (especially, *GNG11*, *RGS6*) that are preferentially expressed within the sinoatrial node underlines the genetic basis that potentially underlies the association between HRV and AF. In short, these genes may affect acetylcholine release of the vagal nerves within the sinoatrial node and thereby influence HRV [16]. More specifically, *GNG11* codes for the γ11 subunit of the heterotrimeric G-protein complex Gαβγ and may cause a decreased expression of this subunit [16]. This lower availability of this subunit may then reduce Gβγ induced GIRK activation. This potentially blunts heart rate changes caused by oscillatory changes in cardiac vagal activity, ultimately decreasing HRV [16]. Furthermore, *RGS6* regulates the heterotrimeric G-protein complex signaling type 6 and may increase its availability. This leads to a decreased GIRK activation and potentially blunts the effects in cardiac vagal activation, and may thereby decrease HRV [16]. Subsequently, it has been suggested that sinus node disease (SND) may cause AF by promoting atrial extrasystoles, and re-entry [40, 41]. Atrial extrasystoles may occur during the slow atrial cycle in the presence of SND. Atrial extrasystoles are mostly followed by a compensatory pause. The pause may then be prolonged which allows other atrial ectopic activity to arise which possibly triggers AF [40]. Early premature beats that originate from areas other than the sinus node may result in conduction block and initiate re-entry, which may be a mechanism underlying AF [40]. Furthermore, stenosis in the sinus nodal artery is also common in patients with AF which implies that ischemic damage to the sinus node alone without atrial fibrosis, stretch or muscle loss may result in AF [40]. Overall, a combination of atrial extrasystoles, re-entry, and ischemia to the sinus node are mechanisms by which SND may cause and promote AF.

We investigated the longitudinal measures of HRV during a long follow-up time in relation to new-onset AF. Taking into account repeated measurements of HRV in relation to new-onset AF may provide more insight and prognostic information over a single baseline measurement that has been done by most of the previous studies [5–10, 13]. Longitudinal measures of HRV during follow-up were associated with an increased risk of incident AF, especially among women. These findings extend previous evidence by simultaneously evaluating the repeated measurements of uncorrected and corrected HRV, heart rate, and sex differences while investigating the link between HRV and AF [5–10, 13]. To some extent our findings support the association between heart rate and AF that has been previously reported in observational, [6, 7, 42] and Mendelian randomization studies [43]. However, we only found a significant association for heart rate in association with AF among women. One potential explanation could be differences in sex hormones. It has been demonstrated that an acute ovarian hormone withdrawal induced by oophorectomy leads to decline in different measures of HRV (SDNN, RMSSD), and an increase in heart rate in women [44]. The same study also showed that estrogen replacement therapy for three months within the oophorectomized women restored the HRV and heart rate to a pre-surgery level [44]. This might explain why uncorrected HRV and heart rate were only associated with incident AF in women, and not in men, in our study. We further hypothesize that competing risk of death is a possible explanation for the observed sex differences. AF is strongly associated with age, [1–3] so it is likely that men die of other (cardiovascular) diseases before development of AF. This hypothesis was supported by our competing risk analyses which showed that SDNN, RMSSD, RMSSDc, and heart rate were significantly associated with mortality, especially among men. Nevertheless, we found a higher incidence of AF in men, than in women in our study.

Our MR approach sheds light on the causality of the association between HRV and AF. Our effect estimates were more or less in line with previous observational studies. However, we were unable to assess the association between SDNNc, RMSSDc and AF, since not enough instrumental variables for SDNNc and RMSSDc were available to be used for the MR analyses. Future GWAS with a larger sample size could identify new additional genetic variants that could be used to assess the association between heart rate corrected HRV and AF. This could be of importance, because of the strong inverse association that exists between HRV and heart rate [24, 25]. This relation is further underlined by Nolte et al. who showed attenuation in the HRV SNP associations when they corrected for heart rate [16]. This might imply that uncorrected measures of HRV may be, in part, confounded by heart rate. Although, we showed that heart rate itself was not significantly associated with new-onset AF (except in women), we cannot rule out the possibility that heart rate is the overall determining factor instead of HRV after all. Since our uncorrected measures of HRV were indeed not significantly associated with new-onset AF. Further, as heart rate is also associated with AF and cardiovascular mortality proper adjustment for heart rate is of importance [26, 43, 45, 46]. However, excluding a genetic variant that was also associated with heart rate, a potential confounder or horizontal mediator, did not substantially change our MR results. Future studies on HRV measures corrected for heart rate could further aid in elucidating the exact mechanisms underlying HRV and AF.

The major strengths of this study are its population-based nature, large sample size with detailed information on cardiovascular risk factors, meticulous adjudication of incident AF and long follow-up time, multiple sensitivity analyses including complete-case analyses, excluding prevalent and incident CHD prior to AF diagnosis, use of competing risk analyses to compute cause-specific hazards, and use of large-scale GWAS summary statistics. The availability of repeated measurements for different HRV measures during follow-up also enabled us to investigate longitudinal measures of HRV in association with new-onset AF in a joint modeling approach which may provide more insight and give more prognostic information over a single baseline measurement. Moreover, using a MR approach we were able to gain more insight in the complex interaction between HRV and AF and to avoid certain biases that are more common in traditional observational epidemiological studies, such as residual confounding and reverse causation [15]. However, our study also has some limitations that should be taken into consideration. Our HRV measures were based on 10-s ECGs, although HRV guidelines recommend that HRV measures are based on preferably 5-min or 24-h ECG recordings [22]. Nevertheless, 10-s ECGs are more commonly performed in healthcare, are cheaper, are faster, and thereby more patient friendly than longer ECG recordings. Additionally, HRV measures from 10-s ECGs have already been associated with left ventricular function, [47] heart failure, [47, 48] cardiac-[49] and all-cause mortality [50]. Additionally, other studies that investigated the reliability of 10-s ECGs in comparison to 5-min ECGs to assess HRV showed that 10-s ECGs are also a reliable tool for HRV risk assessment, in particular within population-based studies [51, 52]. We could not distinguish between paroxysmal, persistent, long-term persistent, and permanent AF as Holter monitoring has not been done in this large population-based cohort. In our MR analyses, we cannot rule out unobserved horizontal pleiotropy, although we tried to address horizontal pleiotropy using multiple MR sensitivity analyses, such as MR-Egger, WME, MR-PRESSO, and sensitivity plots, to identify and correct for horizontal pleiotropy. Additionally, not enough sex-stratified SNPs were available in the publically available genetic dataset to perform the MR for men and women separately. Furthermore, there was partial overlap in the samples that were used to obtain the genetic instruments which may cause bias toward observational findings [33]. However, the potential bias was probably negligible given that the maximum potential overlap was 2.1%. Finally, our findings may not be generalizable to younger populations and other ethnicities, as our analysis included mainly older participants from European descent.

In conclusion, longitudinal measures of SDNN, RMSSD, but not SDNNc, RMSSDc, and heart rate, were significantly associated with new-onset AF. In sex-stratified analyses, RMSSD among men and SDNN, RMSSD, and heart rate among women were significantly associated with new-onset AF. MR analysis confirmed the complex association between HRV and AF that has been indicated by our and previous observational studies. These findings indicate that measures to modulate HRV might prevent AF in the general population, especially among women, but future MR studies that investigate the causality between heart rate corrected measures of HRV and AF are warranted.

## Supplementary Information

Below is the link to the electronic supplementary material.

Supplementary file1 (DOCX 45 kb)

## Data Availability

For the longitudinal study, data can be obtained upon request. Requests should be directed toward the management team of the Rotterdam Study (secretariat.epi@erasmusmc.nl), which has a protocol for approving data requests. Because of restrictions based on privacy regulations and informed consent of the participants, data cannot be made freely available in a public repository. For the Mendelian randomization study, genetic variants associated with HRV, used as instrumental variables for the MR analyses, were retrieved from publically available summary statistics from the 2 used GWAS.
