# Supplementary material for: Heart rate variability and atrial fibrillation in the general population: a longitudinal and Mendelian randomization study
Source: Clin Res Cardiol. 2022 Aug 13;112(6):747–58. doi: 10.1007/s00392-022-02072-5 (PMC10241681; doi:10.1007/s00392-022-02072-5)
Supplement: Supplementary file 1 — Supplementary file1 (DOCX 45 kb) [file 392_2022_2072_MOESM1_ESM.docx]

**SUPPLEMENTARY MATERIAL**

**Methods S1. Assessment of cardiovascular risk factors**

**Methods S2. Joint model analyses**

**Methods S3. Mendelian randomization analyses**

**Methods S4. Study population of the genome-wide association study from which the genetic instruments for heart rate variability were obtained**

**Methods S5. Study population of the genome-wide association study from which the genetic instruments for atrial fibrillation were obtained**

**Results S1. Joint model sensitivity analyses**

**Results S2. Mendelian randomization sensitivity analyses**

**Table S1. Association between longitudinal measures of heart rate variability with the risk of new-onset atrial fibrillation in the total study population and stratified by sex with non-imputed data**

**Table S2. Association between longitudinal measures of heart rate variability with the risk of new-onset atrial fibrillation in the total study population and stratified by sex with exclusion of prevalent and incident coronary heart disease cases before atrial fibrillation**

**Table S3. Association between longitudinal measures of heart rate variability with the risk of mortality in the total study population and stratified by sex**

**Table S4. Effect estimates for the associations of the genetic variants with SDNN, RMSSD and atrial fibrillation**

**Methods S1. Assessment of cardiovascular risk factors**

All participants responded to comprehensive questionnaires at baseline to evaluate their current health status, medical history, medication use, and lifestyle. In addition, the participants were interviewed at home by trained interviewers, underwent more extensive clinical examination, and laboratory assessments at the research center.(19, 20)

Standardized measurements of height (in m) and weight (in kg) were performed and body mass index (BMI) was calculated as weight divided by height squared. Serum total and high-density lipoprotein (HDL) cholesterol were measured with an automated enzymatic method. Blood pressure was measured twice at the right upper arm with a random zero mercury sphygmomanometer in the sitting position. Systolic and diastolic blood pressures were calculated as the mean of the 2 consecutive measurements. Hypertension was defined as a systolic blood pressure of ≥140 mmHg or a diastolic blood pressure ≥90 mmHg or the use of blood pressure lowering drugs prescribed for hypertension.(3) Smoking status was derived from baseline questionnaires and was categorized into never, former, and current smokers. Diabetes mellitus (DM) was defined as fasting serum glucose levels ≥7.0 mmol/L (126 mg/dL) (or non-fasting serum glucose levels ≥11.1 mmol/L (200 mg/dL) if fasting samples were unavailable) or the use of antidiabetic therapy. The assessment and definition of coronary heart disease (CHD), and heart failure (HF) has been described in detail elsewhere.(20) Left ventricular hypertrophy (LVH) was diagnosed using MEANS with an algorithm that takes into account QRS voltages, with an age-dependent correction and repolarization. Medication use was derived from baseline questionnaires, pharmacy data and was categorized and defined according to the World Health Organization Anatomical Therapeutic Chemical (WHO ATC) classifications. Specifically, cardiac medication, antihypertensive medication, use of beta blockers, use of calcium blockers, and lipid lowering medication were defined according to the WHO ATC categories c01, c02, c07, c08, and c10 respectively.

**Methods S2. Joint model analyses**

The baseline characteristics of the study population are presented as mean with standard deviation (SD) or number (n) with percentages as appropriate. Differences between men and women were examined by Student’s T-test (normal distribution) or the Mann Whitney U-test (skewed distribution) for continuous variables and Chi-Square test for categorical variables. The distributions of the different HRV measures and heart rate were skewed. Therefore, a natural logarithmic transformation was used to obtain a normal distribution.

Competing risk analyses were performed using joint models for longitudinal and time to event data. To investigate the association between longitudinal measures of HRV with the risk of new-onset AF with mortality as a competing event. Cause-specific hazard ratios (HRs) with their 95% confidence intervals (CIs) were calculated to quantify the associations. First, linear mixed effects models were used to model the longitudinal measures of HRV and to account for the correlation of repeated measures. The outcome of interest in each mixed effects model was either SDNN, SDNNc, RMSSD, RMSSDc, and heart rate with up to 5 repeated measurements during follow-up. Time was measured in years after baseline and cardiovascular risk factors/covariates were treated as fixed effects in all models. The models investigating the longitudinal measures of SDNN, SDNNc, RMSSD, RMSSDc, and heart rate included a random intercept and slope, and an unstructured covariance matrix. Next, the results from the mixed effects models and Cox models were combined. This was done using the repeated measures for either SDNN, SDNNc, RMSSD, RMSSDc, and heart rate from the linear mixed effects models as a time-dependent covariate in the Cox models.(27)

The analyses were performed in the total study population and for men and women separately. Additionally, we reported the p-values of sex interaction from the joint model. All models (mixed- and survival models) were adjusted for age, sex (if applicable), and cohort (model 1), and additionally for cardiovascular risk factors including BMI, total cholesterol, HDL cholesterol, hypertension, smoking status, history of DM, history of CHD, history of HF, LVH on the ECG, use of cardiac medication, use of beta blockers, use of calcium blockers, and use of lipid lowering medication (model 2). Time was measured in years after baseline and the variables from model 1 and 2 were treated as covariates in the subsequent models. Missing values of any covariates were imputed under the assumption of missing at random using the “mice” package in R.(28) For imputation, all available data were used to generate 1 imputed dataset. Missing values for various covariates were as follows: BMI (2.0%), total cholesterol (6.9%), HDL cholesterol (6.8%), systolic blood pressure (1.2%), diastolic blood pressure (1.2%), smoking status (1.6%), history of CHD (2.4%), history of HF (0.1%), LVH on the ECG (9.5%), use of cardiac medication (5.3%), use of antihypertensive medication (5.4%), use of beta blockers (5.3%), use of calcium blockers (5.3%), and use of lipid lowering medication (5.3%).

As sensitivity analyses, we assessed the associations using complete-case analyses based on non-imputed data. Moreover, we assessed the associations after exclusion of participants with prevalent CHD and incident CHD before the onset of AF to assess if this would attenuate our original findings. Finally, we calculated the cause-specific HRs for mortality to evaluate the competing risk of mortality with incident AF.

**Methods S3. Mendelian randomization analyses**

MR enables assessment of causality by using information on genetic variants. As genetic variants are randomly distributed from parents to offspring at conception, this random distribution of alleles is thereby not influenced by confounding or disease status (reverse causation).(15) We conducted two-sample MR analyses to examine the potential causal association between HRV and AF. MR analyses requires 3 assumptions to provide valid causal estimates.(15) The first assumption is that the genetic variant is strongly associated with the corresponding exposure (HRV). The second assumption is that the genetic variant only affects the outcome (AF) through its effect on the exposure (HRV) and the third assumption is that the genetic variant is not associated with any confounders of the exposure-outcome (HRV-AF) association.

We used the TwoSampleMR package(30, 34, 35) to estimate the effects of the individual genetic instruments using inverse-variance weighted (IVW) analyses.(29) The IVW method is a combined estimate of the Wald ratios from all the individual genetic variants. This means that the IVW method is a weighted mean estimate of the effect of a genetically determined HRV on AF risk. Moreover, we used the random effect IVW method to account for possible heterogeneity between the genetic variants and to relax the no horizontal pleiotropy assumption. MR estimates are presented as odds ratios (ORs) with corresponding 95% confidence intervals (CIs).

Furthermore, the F statistic, a strength measure for the genetic instruments, was calculated for each genetic instrument and a value of F>10 was considered as sufficient strength.(30) We only included genetic variants with F>10 to limit weak instrument bias and to meet the first MR assumption. Furthermore, if the genetic variants show horizontal pleiotropic effects that influence the outcome through pathways other than through the exposure this may lead to biased MR estimates. Therefore, in an attempt to meet the second and third MR assumption, we performed additional analyses including weighted median estimator (WME), MR‐Egger and Mendelian randomization pleiotropy residual sum and outlier (MR-PRESSO) to account and test for horizontal pleiotropy.(31, 32) We used the weighted median estimator (WME) method, which gives a weighted median effect of genetically determined HRV on AF risk.(15) The WME method assumes that only half the genetic variants need to be valid genetic instruments (so, no violation of the 3 MR assumptions for half of the genetic variants). The MR-Egger intercept will not significantly deviate from zero and the MR-Egger slope will be in line with the IVW and WME in the absence of horizontal pleiotropy. In short, if we obtain similar effect estimates from the IVW, WME, MR-Egger slope this would indicate that the MR results are robust.(32) In addition, we used MR-PRESSO to identify horizontal pleiotropic outliers and to provide an outlier-corrected estimate.(31) Moreover, sensitivity plots such as scatter plots were also used to identify potential outliers, if needed. Heterogeneity between the genetic variants could also be an indication of horizontal pleiotropy and we therefore tested heterogeneity using Cochran’s Q test. Furthermore, we excluded genetic variants that were also associated with heart rate, a potential confounder, horizontal mediator of the exposure-outcome association, since this may bias our estimates. Finally, we determined the potential overlap between the study samples that were used to identify the genetic variants, because sample overlap in two-sample MR analyses might potentially cause bias towards the observational results (**Methods S4** and **Methods S5**).(33)

**Methods S4. Study population of the genome-wide association study from which the genetic instruments for heart rate variability were obtained**

| **N of studies** | **N of participants** | **Ethnicity** | **Phenotype definition** |
| --- | --- | --- | --- |
| 29 | Discovery analysis in 28,700 individuals. | European (100%). | HRV traits were extracted from the IBI time series preferably based on 2-10 min periods of ECG in a standardized setting, at rest and in a sitting/supine position. |
| Abbreviations of the included studies ^*^ | | | |
| **ARIC EA**, CARLA, **CHS**, **FHS**, **FINCAVAS**, FINGESTURE, FLEMNGHO-EPOGH, GenR, GTR, **KORA S4**, Lifelines, **MESA**, MRC NSHD, MRS, NESDA, NFBC 1966, NTR, **PIVUS**, **PREVEND**, **RS I**, **RS II**, TRAILS-CC, TRAILS-Pop, UCSD TWINS, **ULSAM**, WHI CT-Garnet, WHI CT-MOPMAP, WHII, YFS. | | | |

The data with the potential overlapping samples with GWAS for atrial fibrillation are marked in **bold**. The exact extent of overlap could not be determined due to unavailability of individual level data, however considering the names of the included studies in the GWAS. The potential overlap was possible between HRV and AF for 21,617 individuals.

^*^ For further details, please see Table S2 of the GWAS from Nolte et al.(16)

**Methods S5. Study population of the genome-wide association study from which the genetic instruments for atrial fibrillation were obtained**

| **N of studies** | **N of participants** | **Ethnicity** | **Phenotype definition** |
| --- | --- | --- | --- |
| 40 | Discovery analysis in 1,030,836 individuals.  AF: 60,620 cases, and 970,216 controls. | Mainly European (>98%). | AF cases were defined as patients with ICD-9: 427.31 or ICD-10: I48. |
| Abbreviations of the included studies ^* †^ | | | |
| AGES, ANGES, ARIC AA, **ARIC EA**, Beat-AF, Biobank Japan, BioMe-Omni AA, BioMe Omni-EA, BioMe-Omni HA, BioVU, CCAF, **CHS** **AA**, **CHS EA**, COROGENE, deCODE, DiscovEHR, **FHS**, **FINCAVAS**, GS:SFHS, HUNT, **KORA**, LURIC, MDC-CC/MDCS, **MESA**, MGH AF study, MGH CAMP, MGI, **PIVUS**, **PREVEND**, PROSPER, **RS I**, **RS II**, RS III, SHIP, SPHFC, TwinGene, UK Biobank, **ULSAM**, WGHS, WTCCC2 Munich. | | | |

The data with the potential overlapping samples with GWAS for HRV are marked in **bold**. The exact extent of overlap could not be determined due to unavailability of individual level data, however considering the names of the included studies in the GWAS. The potential overlap was possible between HRV and AF for 21,617 individuals.

^*^ For further details, please see Table S1 of the original GWAS from Nielsen et al.(17)

^†^ For further details, please see Table S1 of the original GWAS from Christophersen et al.(18)

**Results S1. Joint model sensitivity analyses**

Our sensitivity analyses showed that the results after imputation did not differ substantially from the complete-case analyses (**Table S1**). Moreover, excluding participants with prevalent and incident CHD (prior to incident AF) from the analyses did not change the original results noteworthy (**Table S2**). Lastly, in our competing risk analyses, SDNN, RMSSD, RMSSDc, and heart rate were all significantly associated with mortality in both model 1 and 2 which confirms that mortality is a potential competing risk for incident AF, especially among men (**Table S3**).

**Results S2. Mendelian randomization sensitivity analyses**

The estimates of the WME and MR-Egger slope method were in line with the IVW method after correcting for outliers using MR-PRESSO and additionally examining the sensitivity plots such as the scatter plots during the analyses. In addition, we found no evidence for the presence of directional horizontal pleiotropy after removal of outliers using the MR-Egger intercept (p-value for SDNN: 7.55x10^-01^, and p-value for RMSSD: 7.63x10^-01^) and MR-PRESSO (p-value for SDNN: 9.92x10^-01^, and p-value for RMSSD: 9.08x10^-01^). Moreover, we found no evidence for heterogeneity between the genetic variants using Cochran’s Q test (p-value of the IVW method for SDNN: 9.82x10^-01^, and p-value of the IVW method for RMSSD: 9.26x10^-01^). See **Table 3** for more detailed information. Similar results were observed when we excluded a genetic variant in our analyses that was also associated with heart rate which could be a potential confounder and horizontal mediator (data not shown). Determination of the exact extent of sample overlap between the 2 study samples was not possible due to unavailability of individual level data. However, considering the study names included in both GWAS, there was potential overlap between HRV and AF for 21,617 individuals and to what extent this might have biased our results is uncertain. However, given the sample size of the largest GWAS which included 1,030,836 participants,(17) the potential overlap was probably negligible (21,617/1,030,836=2.10%) See **Methods** S**4.** and **Methods S5.** for detailed information on both GWAS.

**Table S1. Association between longitudinal measures of heart rate variability with the risk of new-onset atrial fibrillation in the total study population and stratified by sex with non-imputed data**

|  | **Total study population** | | **Men** | | **Women** | |
| --- | --- | --- | --- | --- | --- | --- |
| **Heart rate variability measures** | **Cause-specific HR (95% CI)** | | | | | |
|  | **Model 1** ^*^ | **Model 2** ^†^ | **Model 1** ^*^ | **Model 2** ^†^ | **Model 1** ^*^ | **Model 2** ^†^ |
| **SDNN** ^‡^ | 1.19 (0.98-1.45), p=0.0808 | 1.22 (0.97-1.50), p=0.0798 | 1.00 (0.79-1.28), p=0.9539 | 1.02 (0.78-1.32), p=0.8994 | 1.59 (1.12-2.23), p=0.0101 | 1.50 (1.10-2.02), p=0.0090 |
| **SDNNc** ^‡^ | 0.99 (0.84-1.17), p=0.9260 | 1.06 (0.87-1.26), p=0.4903 | 0.95 (0.77-1.18), p=0.6448 | 0.98 (0.79-1.19), p=0.8509 | 1.05 (0.80-1.37), p=0.6977 | 1.13 (0.88-1.46), p=0.3448 |
| **RMSSD** ^‡^ | 1.35 (1.16-1.59), p=0.0001 | 1.32 (1.11-1.58), p=0.0026 | 1.11 (0.90-1.38), p=0.3262 | 1.10 (0.88-1.37), p=0.3813 | 1.76 (1.34-2.28), p=<0.0001 | 1.60 (1.26-2.03), p=0.0001 |
| **RMSSDc** ^‡^ | 1.05 (0.92-1.21), p=0.4557 | 1.09 (0.94-1.25), p=0.2119 | 1.02 (0.87-1.20), p=0.8034 | 1.04 (0.87-1.22), p=0.6484 | 1.11 (0.88-1.38), p=0.3193 | 1.15 (0.95-1.40), p=0.1578 |
| **Heart rate** ^‡^ | 1.41 (0.80-2.43), p=0.2511 | 1.09 (0.55-2.03), p=0.7599 | 0.68 (0.36-1.29), p=0.2459 | 0.65 (0.34-1.25), p=0.1986 | 1.91 (0.93-3.68), p=0.0806 | 1.35 (0.69-2.60), p=0.3777 |

**Abbreviations:** CI, confidence interval; HR, hazard ratio; RMSSD, root mean square of successive RR interval differences; RMSSDc, root mean square of successive RR interval differences corrected for heart rate; SDNN, standard deviation of normal to normal RR intervals; SDNNc, standard deviation of normal to normal RR intervals corrected for heart rate.
^*^ Adjusted for age, sex (if applicable), and cohort.
^†^ Adjusted for age, sex (if applicable), cohort, body mass index, total cholesterol, high-density lipoprotein cholesterol, hypertension, smoking status, history of diabetes mellitus, history of coronary heart disease, history of heart failure, left ventricular hypertrophy on the electrocardiogram, use of cardiac medication, use of beta blockers, use of calcium blockers, and use of lipid lowering medication use.
^‡^ Hazard ratios represent 1 unit increase of ln(SDNN), ln(SDNNc), ln(RMSSD), ln(RMSSDc), and 1 unit decrease of ln(heart rate) with the risk of new-onset atrial fibrillation.

**Table S2. Association between longitudinal measures of heart rate variability with the risk of new-onset atrial fibrillation in the total study population and stratified by sex with exclusion of prevalent and incident coronary heart disease cases before atrial fibrillation**

|  | **Total study population** | | **Men** | | **Women** | |
| --- | --- | --- | --- | --- | --- | --- |
| **Heart rate variability measures** | **Cause-specific HR (95% CI)** | | | | | |
|  | **Model 1** ^*^ | **Model 2** ^†^ | **Model 1** ^*^ | **Model 2** ^†^ | **Model 1** ^*^ | **Model 2** ^†^ |
| **SDNN** ^‡^ | 1.34 (1.11-1.62), p=0.0012 | 1.34 (1.11-1.60), p=0.0010 | 1.26 (0.97-1.62), p=0.0790 | 1.26 (0.97-1.65), p=0.0766 | 1.47 (1.11-1.99), p=0.0065 | 1.32 (0.99-1.74), p=0.0612 |
| **SDNNc** ^‡^ | 1.02 (0.86-1.19), p=0.8064 | 1.05 (0.91-1.22), p=0.4983 | 1.12 (0.89-1.37), p=0.3128 | 1.11 (0.88-1.39), p=0.3562 | 0.94 (0.72-1.23), p=0.6053 | 0.96 (0.73-1.22), p=0.7967 |
| **RMSSD** ^‡^ | 1.48 (1.27-1.73), p<0.0001 | 1.43 (1.23-1.66), p<0.0001 | 1.37 (1.09-1.70), p=0.0070 | 1.35 (1.10-1.67), p=0.0045 | 1.65 (1.30-2.05), p<0.0001 | 1.47 (1.15-1.87), p=0.0008 |
| **RMSSDc** ^‡^ | 1.09 (0.96-1.23), p=0.1935 | 1.09 (0.97-1.22), p=0.1506 | 1.16 (0.97-1.37), p=0.0943 | 1.15 (0.97-1.38), p=0.1122 | 1.00 (0.84-1.19), p=0.9921 | 1.00 (0.83-1.20), p=0.9713 |
| **Heart rate** ^‡^ | 1.73 (1.10-2.67), p=0.0223 | 1.56 (0.91-2.55), p=0.0997 | 0.68 (0.36-1.32), p=0.2610 | 0.77 (0.37-1.52), p=0.4577 | 2.80 (1.24-5.68), p=0.0165 | 1.75 (0.93-3.24), p=0.0827 |

**Abbreviations:** CI, confidence interval; HR, hazard ratio; RMSSD, root mean square of successive RR interval differences; RMSSDc, root mean square of successive RR interval differences corrected for heart rate; SDNN, standard deviation of normal to normal RR intervals; SDNNc, standard deviation of normal to normal RR intervals corrected for heart rate.
^*^ Adjusted for age, sex (if applicable), and cohort.
^†^ Adjusted for age, sex (if applicable), cohort, body mass index, total cholesterol, high-density lipoprotein cholesterol, hypertension, smoking status, history of diabetes mellitus, history of coronary heart disease, history of heart failure, left ventricular hypertrophy on the electrocardiogram, use of cardiac medication, use of beta blockers, use of calcium blockers, and use of lipid lowering medication use.
^‡^ Hazard ratios represent 1 unit increase of ln(SDNN), ln(SDNNc), ln(RMSSD), ln(RMSSDc), and 1 unit decrease of ln(heart rate) with the risk of new-onset atrial fibrillation.

**Table S3. Association between longitudinal measures of heart rate variability with the risk of mortality in the total study population and stratified by sex**

|  | **Total study population** | | **Men** | | **Women** | |
| --- | --- | --- | --- | --- | --- | --- |
| **Heart rate variability measures** | **Cause-specific HR (95% CI)** | | | | | |
|  | **Model 1** ^*^ | **Model 2** ^†^ | **Model 1** ^*^ | **Model 2** ^†^ | **Model 1** ^*^ | **Model 2** ^†^ |
| **SDNN** ^‡^ | 0.82 (0.73-0.91), p<0.0001 | 0.82 (0.74-0.91), p<0.0001 | 0.74 (0.64-0.85), p<0.0001 | 0.76 (0.66-0.87), p=0.0004 | 0.89 (0.76-1.03), p=0.1262 | 0.87 (0.74-1.02), p=0.0848 |
| **SDNNc** ^‡^ | 1.00 (0.92-1.09), p=0.9970 | 1.01 (0.92-1.10), p=0.9046 | 1.02 (0.92-1.14), p=0.6745 | 1.01 (0.90-1.13), p=0.8441 | 0.97 (0.85-1.10), p=0.6114 | 0.98 (0.86-1.11), p=0.7008 |
| **RMSSD** ^‡^ | 0.91 (0.83-0.99), p=0.0218 | 0.89 (0.82-0.98), p=0.0119 | 0.82 (0.73-0.93), p=0.0011 | 0.83 (0.74-0.93), p=0.0019 | 1.00 (0.89-1.13), p=0.9638 | 0.97 (0.85-1.10), p=0.6155 |
| **RMSSDc** ^‡^ | 1.09 (1.02-1.16), p=0.0079 | 1.08 (1.01-1.15), p=0.0234 | 1.13 (1.03-1.23), p=0.0074 | 1.10 (1.01-1.20), p=0.0287 | 1.05 (0.96-1.15), p=0.3093 | 1.05 (0.95-1.16), p=0.3291 |
| **Heart rate** ^‡^ | 0.47 (0.34-0.65), p<0.0001 | 0.45 (0.33-0.62), p<0.0001 | 0.28 (0.19-0.43), p<0.0001 | 0.31 (0.21-0.48), p<0.0001 | 0.90 (0.59-1.38), p=0.6154 | 0.71 (0.45-1.10), p=0.1204 |

**Abbreviations:** CI, confidence interval; HR, hazard ratio; RMSSD, root mean square of successive RR interval differences; RMSSDc, root mean square of successive RR interval differences corrected for heart rate; SDNN, standard deviation of normal to normal RR intervals; SDNNc, standard deviation of normal to normal RR intervals corrected for heart rate.
^*^ Adjusted for age, sex (if applicable), and cohort.
^†^ Adjusted for age, sex (if applicable), cohort, body mass index, total cholesterol, high-density lipoprotein cholesterol, hypertension, smoking status, history of diabetes mellitus, history of coronary heart disease, history of heart failure, left ventricular hypertrophy on the electrocardiogram, use of cardiac medication, use of beta blockers, use of calcium blockers, and use of lipid lowering medication use.
^‡^ Hazard ratios represent 1 unit increase of ln(SDNN), ln(SDNNc), ln(RMSSD), ln(RMSSDc), and 1 unit decrease of ln(heart rate) with the risk of new-onset atrial fibrillation.

**Table S4. Effect estimates for the associations of the genetic variants with SDNN, RMSSD and atrial fibrillation**

|  | | | | **Exposure effect estimates** | | | | | | | | **Outcome effect estimates** | | | | | |
| --- | --- | --- | --- | --- | --- | --- | --- | --- | --- | --- | --- | --- | --- | --- | --- | --- | --- |
| **SNP** | **Chr** | **Pos** | **Gene** | **Trait** | **EA** | **OA** | **EAF** | **Beta** | **SE** | **Pval** | **N** | **Trait** | **EAF** | **Beta** | **SE** | **Pval** | **N** |
| rs236349 | 6 | 36928543 | PPIL1 | SDNN | G | A | 0.650985 | -0.032710 | 0.003155 | 3.70x10^-25^ | 51,379 | AF | 0.650900 | -0.012200 | 0.007000 | 8.01x10^-02^ | 1,030,836 |
| rs4262 | 7 | 93389364 | GNG11 | SDNN | C | T | 0.389763 | -0.027710 | 0.003294 | 4.26x10^-17^ | 49,005 | AF | 0.422700 | -0.013600 | 0.006800 | 4.63x10^-02^ | 1,030,836 |
| rs36423 | 14 | 71422955 | RGS6 | SDNN | T | G | 0.129193 | -0.032977 | 0.004580 | 6.25x10^-13^ | 48,182 | AF | 0.141600 | -0.018600 | 0.010300 | 6.96x10^-02^ | 1,030,836 |
| rs4899412 | 14 | 71534015 | RGS6 | SDNN | T | C | 0.252324 | -0.025882 | 0.003548 | 3.13x10^-13^ | 48,252 | AF | 0.273200 | -0.011700 | 0.007600 | 1.22x10^-01^ | 1,030,836 |
| rs2529471 | 14 | 71883022 | RGS6 | SDNN | C | A | 0.428286 | -0.021160 | 0.003002 | 1.88x10^-12^ | 49,619 | AF | 0.429000 | -0.012000 | 0.006700 | 7.43x10^-02^ | 1,030,836 |
| rs236349 | 6 | 36928543 | PPIL1 | RMSSD | G | A | 0.654958 | -0.035488 | 0.004264 | 9.10x10^-17^ | 46,795 | AF | 0.650900 | -0.012200 | 0.007000 | 8.01x10^-02^ | 1,030,836 |
| rs180238 | 7 | 93388383 | GNG11 | RMSSD | C | T | 0.333966 | -0.034439 | 0.004273 | 7.99x10^-16^ | 44,420 | AF | 0.350500 | -0.011400 | 0.007100 | 1.08x10^-01^ | 1,030,836 |
| rs7980799 | 12 | 33468257 | SYT10 | RMSSD | A | C | 0.391851 | -0.038742 | 0.004202 | 3.19x10^-20^ | 44,210 | AF | 0.414900 | -0.023300 | 0.006800 | 6.65x10^-04^ | 1,030,836 |
| rs36423 | 14 | 71422955 | RGS6 | RMSSD | T | G | 0.127977 | -0.040464 | 0.006163 | 5.36x10^-11^ | 45,419 | AF | 0.141600 | -0.018600 | 0.010300 | 6.96x10^-02^ | 1,030,836 |
| rs4899412 | 14 | 71534015 | RGS6 | RMSSD | T | C | 0.253558 | -0.028307 | 0.004651 | 1.19x10^-09^ | 45,423 | AF | 0.273200 | -0.011700 | 0.007600 | 1.22x10^-01^ | 1,030,836 |
| rs2529471 | 14 | 71883022 | RGS6 | RMSSD | C | A | 0.424941 | -0.026200 | 0.003934 | 2.83x10^-11^ | 46,790 | AF | 0.429000 | -0.012000 | 0.006700 | 7.43x10^-02^ | 1,030,836 |

**Abbreviations:** SNP: single nucleotide polymorphism, Chr: chromosome, Pos: genomic position, EA: effect allele, OA: other allele, EAF: effect allele frequency, SE: standard error, Pval: p-value, N: sample size, SDNN: standard deviation of normal to normal RR intervals, RMSSD: the root mean square of successive RR interval differences.
